# Novel function of PIWIL1 in neuronal polarization and migration via regulation of microtubule-associated proteins

**DOI:** 10.1186/s13041-015-0131-0

**Published:** 2015-06-24

**Authors:** Ping-ping Zhao, Mao-jin Yao, Si-yuan Chang, Lan-tao Gou, Mo-fang Liu, Zi-long Qiu, Xiao-bing Yuan

**Affiliations:** Institute of Neuroscience and State Key Laboratory of Neuroscience, Shanghai Institutes for Biological Sciences, Chinese Academy of Sciences, Shanghai, 200031 China; Graduate School of Chinese Academy of Sciences, Shanghai, 200031 China; State Key Laboratory of Molecular Biology, Institute of Biochemistry and Cell Biology, Shanghai Institutes for Biological Sciences, Chinese Academy of Sciences, Shanghai, 200031 China; Current Affiliation: Hussman Institute for Autism, Baltimore, MD 21201 USA

**Keywords:** PIWIL1, Radial migration, Polarization, Microtubule-associated proteins

## Abstract

**Background:**

Young neurons in the developing brain establish a polarized morphology for proper migration. The PIWI family of piRNA processing proteins are considered to be restrictively expressed in germline tissues and several types of cancer cells. They play important roles in spermatogenesis, stem cell maintenance, piRNA biogenesis, and transposon silencing. Interestingly a recent study showed that *de novo* mutations of PIWI family members are strongly associated with autism.

**Results:**

Here, we report that PIWI-like 1 (PIWIL1), a PIWI family member known to be essential for the transition of round spermatid into elongated spermatid, plays a role in the polarization and radial migration of newborn neurons in the developing cerebral cortex. Knocking down PIWIL1 in newborn cortical neurons by *in utero* electroporation of specific siRNAs resulted in retardation of the transition of neurons from the multipolar stage to the bipolar stage followed by a defect in their radial migration to the proper destination. Domain analysis showed that both the RNA binding PAZ domain and the RNA processing PIWI domain in PIWIL1 were indispensable for its function in neuronal migration. Furthermore, we found that PIWIL1 unexpectedly regulates the expression of microtubule-associated proteins in cortical neurons.

**Conclusions:**

PIWIL1 regulates neuronal polarization and radial migration partly via modulating the expression of microtubule-associated proteins (MAPs). Our finding of PIWIL1’s function in neuronal development implies conserved functions of molecules participating in morphogenesis of brain and germline tissue and provides a mechanism as to how mutations of PIWI may be associated with autism.

**Electronic supplementary material:**

The online version of this article (doi:10.1186/s13041-015-0131-0) contains supplementary material, which is available to authorized users.

## Background

Newborn neurons produced at the ventricular zone (VZ) in the developing cerebral cortex will first experience a multipolar stage with several minor processes extending from the soma. They will gradually establish a bipolar morphology with one elongated neurite that leads the radial migration of the neuron towards the surface of the cortical plate (CP) [1]. This multipolar–bipolar transition is essential for the maturation and proper migration of newborn neurons. Interestingly, sperm cells exhibit a polarized morphology that is very similar to newborn neurons. These two distinct cell types are both characterized by an elongated microtubule-based structure, an axoneme in sperm and a leading neurite in the migrating neuron, and both play essential roles in cell motility. During spermatogenesis, postmeiotic round spermatids undergo drastic elongation and grow the long axoneme, a morphology change that appears similar to the polarization of newborn neurons in the developing brain. Some molecular mechanisms for establishing the polarized morphology are likely shared at these two different tissues. Knowledge about the morphogenesis of either tissue may assist us in studying the other. This notion is supported by recent findings that several genes previously known to be important for brain morphogenesis, e.g., *LIS1* (lissencephaly type 1) [2, 3] and *CDK5* [4, 5], also play important roles in spermatogenesis [6–8].

The *piwi* genes (P-element-induced wimpy testis) were first identified as important players in the asymmetric division of germline stem cells in *Drosophila* [9]*.* PIWI proteins play essential roles in the biogenesis of a group of small RNAs called piRNAs (PIWI-interacting RNAs) and in the epigenetic silencing of transposable elements [10]. Knockout of *piwi* (*miwi*) in mice causes spermatogenic arrest at the beginning of the round spermatid stage [11], suggesting an essential role for PIWI in spermatid polarization through an unidentified mechanism. Interestingly, a recent whole exome sequencing study involving simplex families that had a child with autism spectrum disorder showed that *de novo* mutations of PIWI family members, especially the PIWIL2 and PIWIL4, are strongly associated with autism [12]. This suggests that PIWIs are involved in the developmental process of the brain. Consistent with this notion, a set of piRNA has been reported to be expressed in mouse hippocampal neurons [13]. A gene profiling study also showed the expression of PIWI mRNA in VZ, subventribular zone-intermediate zone (SVZ-IZ) and CP in mouse embryos [14]. Recently, we also showed the existence of a group of piRNA-like small RNAs, also called repeat associated small interfering RNAs (rasiRNA), in rat cortex (original data at the Gene Expression Omnibus (GEO) database, No. GSE27576) [15], supporting the expression of piRNA biogenesis regulators, including PIWIs, in cortical neurons. In the current study, we observed the expression of PIWI-like 1 (PIWIL1) in developing cerebral cortex of rodents and uncovered a surprising function of this protein in the regulation of neuronal polarization and radial migration. We further discovered that this novel function of PIWIL1 is achieved partly via modulating the expression of microtubule-associated proteins (MAPs).

## Results

### PIWIL1 regulates neuronal radial migration in developing cerebral cortex

We first analyzed the expression of PIWIL1 mRNA in the developing mouse brain using *in situ* hybridization. As shown in Additional file 1: Figure S1A-D, at embryonic day 14.5 (E14.5), PIWIL1 mRNA was expressed at a high level in several regions of the mouse brain including the CP. The VZ/SVZ of the cortex also showed lower levels of PIWIL1 signal. To further clarify whether PIWIL1 is expressed in newborn cortical neurons, we carried out *in utero* electroporation (*IUE*) [16, 17] in rat cortex with plasmids coding for EYFP at E16 and harvested EYFP-positive newborn neurons using fluorescence-aided cell sorter (FACS) at E18 or P0 (Additional file 1: Figure S1E) [18]. As shown in Additional file 1: Figure S1F, semi-quantitative RT-PCR showed that PIWIL1 is indeed expressed in purified newborn neurons. Western blotting analysis of cortical tissues also showed the expression of PIWIL1 in rat cortical tissues, although at a much lower level than is found in the testis (Additional file 1: Figure S1G). There was a gradually decreased expression of PIWIL1 proteins from early embryonic stage to the neonatal stage and later it maintained a low level, correlating with a reduction over time in the level of piRNA-like small RNAs (Additional file 1: Figure S1H and I).

**Fig. 1:**
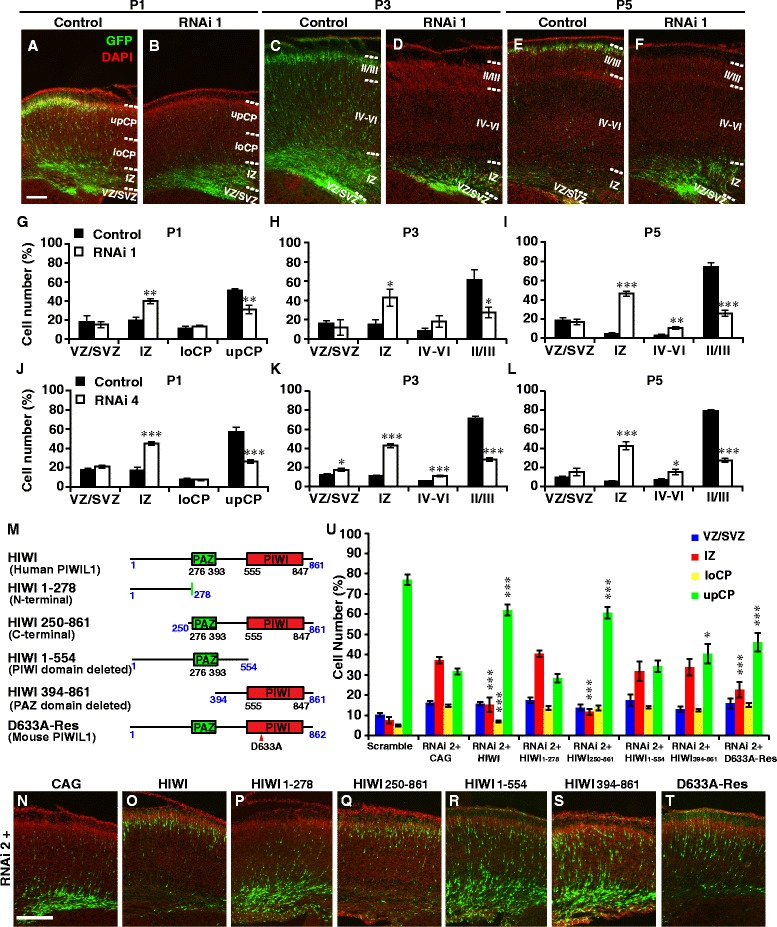
PIWIL1 regulates cortical radial migration. **a**-**f** Effect of knockdown of PIWIL1 in rat cortical neurons by *IUE* with plasmids coding for PIWIL1 siRNA (RNAi 1). Coronal brain sections at different stages were stained with anti-GFP (green) and DAPI (red). **g**-**l** Distribution of labeled cells in brains electroporated with RNAi 1 (**g**-**i**) and RNAi 4 (**j**-**l**) or control constructs (Scramble). **m** Different forms of truncated human PIWIL1 (HIWI) or mutated mouse PIWIL1. **n**-**s**, **u** Only constructs containing both PAZ and PIWI domains rescued the migration. The Scramble control data were from the experiment in Additional file 2: Figure S2E. CAG, pCAG-IRES-GFP vector as control. **t**, **u** Co-electroporation of D633A-Res with RNAi 2 rescued the migration defect. Scale bar, 250 μm. Error bar, SEM. *P < 0.05, **P < 0.01, ***P < 0.001 (Student’s *t*-test)

To test the potential function of PIWIL1 in the development of cortical neurons, we first used *IUE* of plasmids coding for short interference RNAs (siRNAs) targeting PIWIL1 (knockdown efficiency was validated (Additional file 2: Figure S2A and B)) together with plasmids coding for EYFP into cortical progenitor cells in the VZ of rat cortex at E16. At postnatal day 1 (P1), most cells electroporated with the PIWIL1 siRNA (RNAi 1 for rat) failed to migrate into the CP. This migration retardation persisted to later stages of P3 and P5, with aberrantly accumulated cells located in the IZ or deep layers of the CP (upCP, Upper CP; loCP, Lower CP, Fig. 1a-i). Electroporation with another siRNA for rat (RNAi 4, Fig. 1j-l) or effective siRNA for mouse (RNAi 2) (Additional file 2: Figure S2C-E) resulted in a similar migration defect in rat and mouse, respectively. Although overexpression of human PIWIL1 (HIWI) alone did not promote the neuronal migration in mouse cortex (Additional file 2: Figure S2F-H), co-transfection of RNAi 2 along with HIWI, which is highly homologous to mouse PIWIL1 in the protein sequence but could not be targeted by RNAi 2, blocked the knockdown phenotype in mouse cortex (RNAi 2 + CAG v.s. RNAi 2 + HIWI) (Fig. 1n, o, u). Together, these data suggest a specific role of PIWIL1 in the regulation of the radial migration of cortical neurons.

### Both PAZ and PIWI domains are indispensable for proper radial migration

PIWI contains a central PAZ domain (PIWI/argonaute/zwille) that bears a typical single-strand nucleic acid binding motif [10, 19] and a C-terminal PIWI domain that can cut bound RNAs, similar to the RNase H [20]. To test whether the RNA binding and processing activities are required for PIWIL1’s role in neuronal migration, we co-electroporated different truncated HIWI constructs along with the effective siRNA (RNAi 2) in mouse embryos. We found that truncated constructs missing either one of the conserved domains failed to rescue the neuronal migration (Fig. 1m-s, u), suggesting that both PAZ and PIWI domains are indispensable for PIWIL1’s function in neuronal migration. PIWIL1 is known to exhibit the small RNA-guided RNase (slicer) activity, and the D633A single point mutation disrupts this ‘slicer’ activity, leading to LINE1 retrotransposon accumulation and male infertility [21]. We observed that the D633A mutant of PIWIL1 could largely rescue the migration defect caused by PIWIL1 siRNA (Additional file 3: Figure S3, Fig. 1m, t, u), suggesting that the ‘slicer’ activity of PIWIL1 is not essential for its role in neuronal migration.

### PIWIL’s role is mainly in postmitotic neurons

Radial migration of cortical neurons is known to depend on the integrity of radial glial fibers, which provide the scaffold for neuronal migration. The morphology of radial glia fibers did not seem to be changed by PIWIL1 siRNA in rat cortex (Additional file 4: Figure S4). Acute (4-h) and 24-h bromodeoxyuridine (BrdU) incorporation assays showed that knockdown of PIWIL1 did not affect the proliferation of neural progenitor cells in the VZ/SVZ of rat cortex (Additional file 5: Figure S5A, B, E and F). Immunostaining of the neural stem cell marker Sox2 [(sex determining region Y)-box2] 2 days after knockdown of PIWIL1 didn’t show a significant difference in the percentage of the Sox2^+^ population (Additional file 5: Figure S5C and G). The percentage of the Tbr2^+^ transiently amplifying progenitors showed a very slight but significant increase after knockdown of PIWIL1 (Additional file 5: Figure S5D and H). These results suggest that although PIWIL1 may be involved in the proliferation and differentiation of transiently amplifying neural progenitors, it does not play a major regulatory role in these early developmental processes and the severe neuronal migration retardation after knockdown of PIWIL1 may reflect a major function of PIWIL1 in postmitotic neurons.

### PIWIL1 is required for multipolar-bipolar transition of postmitotic neurons

**Fig. 2:**
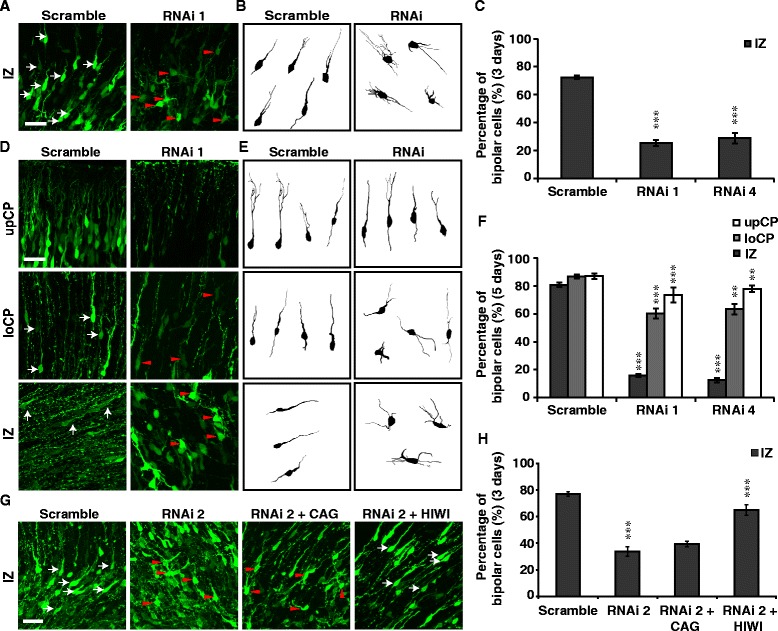
PIWIL1 is required for the multipolar–bipolar transition of postmitotic neurons. **a**, **d** Morphology of labeled neurons in different cortical regions 3 or 5 days post-*IUE* with siRNA 1. **b**, **e** Traces of labeled neurons 3 or 5 days after *IUE* respectively*.* **c, f** Percentage of bipolar cells (white arrows) in different cortical regions. Data are from at least 3 independent *IUE* experiments. **g** Typical morphology of labeled mouse neurons in the IZ 3 days after *IUE* with RNAi 2 or RNAi 2 plus HIWI compared with individual control plasmid. **h** Percentage of bipolar cells at the IZ of electroporated mouse cortex. Scale bar, 30 μm. Error bar, SEM, **P < 0.01, ***P < 0.001 (Student’s *t*-test)

Newborn neurons produced at the VZ of developing cerebral cortex will first experience a multipolar stage with several minor processes extending from the soma and then gradually establish a bipolar morphology with one elongated neurite that leads the migration of the neuron towards the surface of the CP [1]. To analyze whether neuronal polarization defects precedes the retardation of neuronal migration, we traced the morphology of newborn neurons 3 to 5 days after *IUE* of rat embryos and quantified the percentages of bipolar and multipolar cells. Most cells (~70 %) in the IZ had established a bipolar morphology 3 days after *IUE* with Scramble, but less than 30 % of labeled cells had established bipoplar morphology in RNAi 1- or RNAi 4-transfected brains (Fig. 2a-c). Five days after *IUE*, most cells had migrated into the CP in control brains, whereas in RNAi 1- or RNAi 4-transfected brains, most cells were arrested at the IZ and maintained a multipolar morphology (Fig. 2d-f). *IUE* of PIWIL1 siRNA (RNAi 2) in mouse brains caused a similar defect in neuronal polarization, which could be rescued by co-expression of HIWI (Fig. 2g and h).

**Fig. 3:**
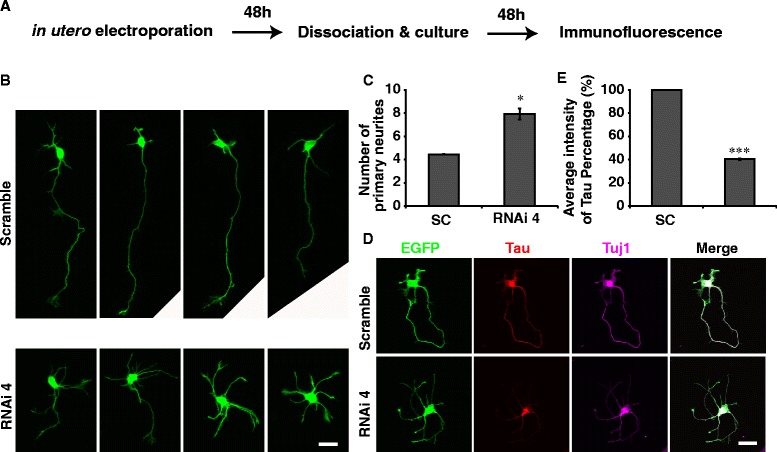
PIWIL1 knockdown impairs polarization of cortical neurons *ex vivo*. **a** Diagram of the *ex vivo* assay. **b**, **c** Average numbers of primary neurites of electroporated cells. **d** Immunostaining: cultured neurons with PIWIL1 knockdown exhibited multipolar morphology and lower levels of Tau but not Tuj1. **e** Average neurites’ fluorescence intensity of Tau in GFP^+^ neurons. Scale bar, 20 μm. Error bar, SEM, *P < 0.05, ***P < 0.001 (Student’s *t*-test)

We further analyzed the effect of PIWI knockdown on neuronal morphology using an *ex vivo* assay, in which newborn neurons derived from electroporated brains were dissociated and cultured for 2 days before morphological analysis (Fig. 3a). We found that neurons with PIWIL1 knockdown showed a multipolar morphology and significantly larger number of primary neurites than that of control neurons (Fig. 3b and c). By labeling the axon with immunostaining of Tau, we observed that neurons in PIWIL1 RNAi group didn’t have a specific axon and unexpectedly showed reduced expression of Tau after 2 days of culture (Fig. 3d). After quantification of the fluorescence intensity, we observed that average fluorescence intensity of Tau staining at the neurite of GFP positive neurons in the RNAi group was significantly lower than that of the control group (Fig. 3e). In contrast, the expression of the pan-neuronal marker Tuj1 was not affected. Taken together, the above results show that PIWIL1 promotes the multipolar–bipolar transition of newborn neurons, which is essential for the proper radial migration of cortical neurons.

### PIWIL1 affects neuronal migration through regulating MAP expression

**Fig. 4:**
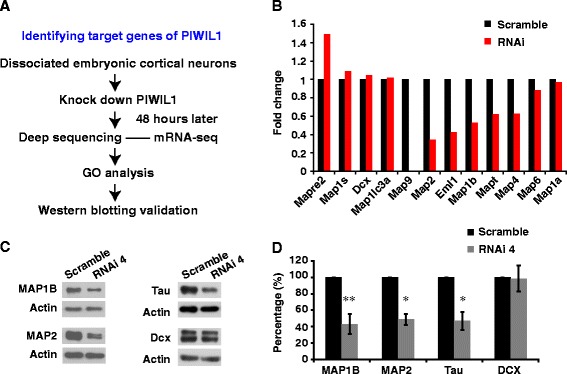
Identification of PIWIL1 target genes. **a** Strategy for identifying target genes. **b** Fold changes of the mRNA levels of several MAPs based on mRNA sequencing results. **c**, **d** Western blots showing the reduction of MAP1B, MAP2, and Tau, but not DCX, by PIWIL1 knockdown in cultured cortical neurons. Error bar, SEM. *P < 0.05, **P < 0.01 (Student’s *t*-test)

To elucidate the target genes of PIWIL1, we used deep sequencing to compare the mRNA profiles of cultured cortical neurons 48 h after they were electroporated with control and PIWIL1 siRNAs (GEO No. GSE48236) (Fig. 4a). With PIWIL1 knockdown, 286 genes were upregulated and 667 genes were downregulated (FDR ≤ 0.05, |log_2_Ratio| ≥ 0.58). Gene ontology (GO) analysis of the group of 667 downregulated genes showed significant enrichment of GO terms related to cytoskeleton organization, especially the microtubule organization (Additional file 6: Table S1). GO analysis of the group of 286 upregulated genes showed significant enrichment of GO terms related to protein transport and establishement of protein localization (Additional file 7: Table S2).

Considering the well-established roles of microtubule dynamics in neuronal polarization, we next focused on the microtubule-associated proteins which may be a potential target of PIWIL1. As shown in Fig. 4b, we found that in the RNAi-treated group the mRNA levels of several microtubule-associated proteins (MAPs), including MAP1B, MAP2, and Tau decreased significantly. Down-regulation of Tau is consistent with a low immunostaining signal of Tau in cultured newborn neurons that had been electroporated with the PIWIL1 siRNA in the *ex vivo assay* shown in Fig. 3d. Western blotting also showed a significant reduction in the levels of MAP1B, MAP2, and Tau, but not doublecortin (DCX) after transfection with PIWIL1 siRNA in cultured cortical neurons (Fig. 4c and d) (Note: Tau image was obtained from the same experiment as that in Fig. 6b).

**Fig. 5:**
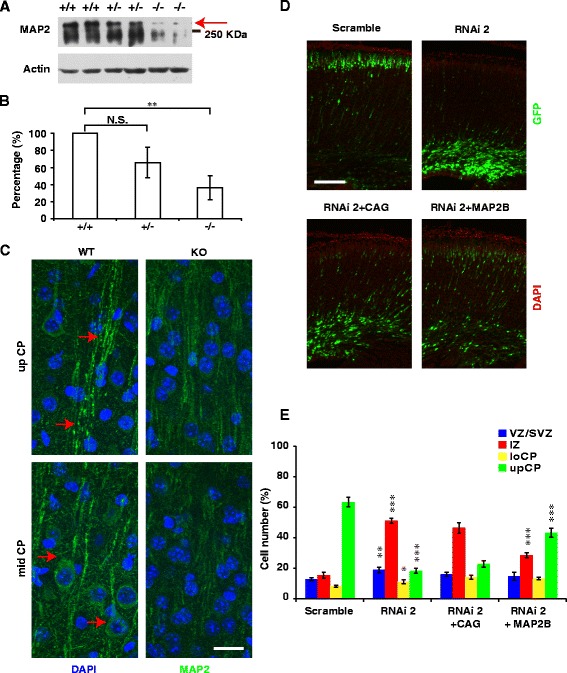
PIWIL1 affects neuronal migration through regulating the expression of MAPs. **a**, **b** MAP2 levels (bands indicated by the arrow) were significantly decreased in the cortical tissue of adult *PIWIL1*-knockout mice (tissues were all from male animals). Statistics analysis was based on 4 technical repeats on pooled samples (*n* = 2). **c** Cortex of *PIWIL1*-knockout mice showed lower level of MAP2 immunofluorescence signal (red arrows indicate MAP2 signal in WT brain). Scale bar, 20 μm. **d**, **e** Co-electroporation of MAP2B with PIWIL1 siRNA largely attenuated the migration defect in mouse brains. Scramble *vs* RNAi 2; RNAi 2 + CAG *vs* RNAi 2 + MAP2B. Scale bar, 150 μm. Error bar, SEM, *P < 0.05, **P < 0.01, ***P < 0.001 (Student’s *t*-test)

The regulation of MAPs by PIWIL1 *in vivo* was further validated using *PIWIL1*-knockout mice. As shown in Fig. 5a-c, in cortical tissues of adult PIWIL1-knockout mice, MAP2 levels were much lower than those in wild-type mice as revealed by both Western blotting (bands indicated by the arrow) and immunostaining. Mice lacking both MAP2 and MAP1B are known to exhibit fiber tract malformations and retarded neuronal migration in the brain [22]. To test whether PIWIL1 regulates neuronal migration via MAPs, we co-transfected the cDNA of one MAP member, MAP2B, together with PIWIL1 siRNA into cortical progenitors using *IUE* in embryonic mouse brains. We found that neuronal migration was largely rescued by co-transfection with MAP2B (Fig. 5d and e).

**Fig. 6:**
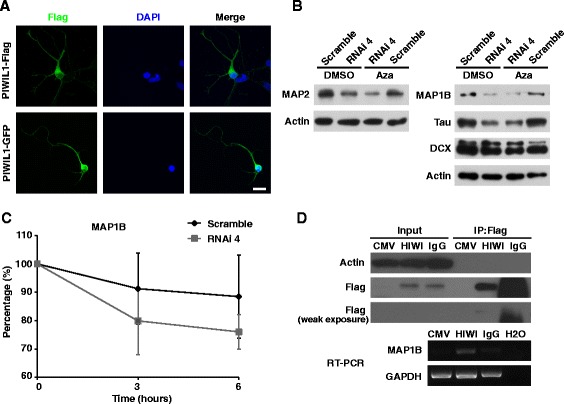
PIWIL1 may regulate MAPs through stabilizing mRNA, but not DNA methylation. **a** Localization of PIWIL1 in neurons. Dissociated cortical neurons were electroporated with Flag or GFP fused PIWIL1 plasmids (green). PIWIL1 was mainly localized to the cytoplasm, Scale bar, 20 μm. **b** Western blotting shows that PIWIL1 knockdown decreased the protein level of MAP1B, MAP2, and Tau, but not DCX. Treatment with the DNA methyltransferase inhibitor 5′AZA (2 μM) did not prevent the reduction of MAPs caused by PIWIL1 siRNA. **c** Neurons were electroporated with Scramble or RNAi plasmid. 48 h later, ActinomycinD (ActD) was added to inhibit transcription for 0, 3 or 6 h. MAP1B mRNA levels were measured by qPCR. The RNA levels at 0 h time point were set as 100 %. Knockdown of PIWIL1 resulted in a faster decay of MAP1B mRNA. Error bar, SEM. **d** Specific interaction between PIWIL1 and the mRNA of MAP1B. Upper panel, cortical neurons were transfected with Flag-fused HIWI or the vector (CMV-3xFlag). The RNA-protein complex was immunoprecipitated (IP) by anti-Flag antibody. The pull-down of HIWI was validated by Western Blotting, with actin as the indicator of equal input. Weak exposure image indicates that band of IgG group is the non-specific signal with lower molecular weight compared to HIWI-Flag. Lower panel, mRNA of MAP1B immunoprecipitated was revealed by semi-quantitative RT-PCR

To further understand how PIWIL1 regulates MAP expression, we first analyzed the subcellular distribution of PIWIL1. Contrary to the nucleic localization of PIWI in Aplysia sensory neurons [23], we observed a predominant cytoplasmic localization of PIWI in cells overexpressing GFP- or Flag-fused PIWIL1 (Fig. 6a). This cytoplasmic distribution of PIWI is similar to what was observed by Lee et al. in hippocampal neurons [13]. Moreover, we found that treating cultured cortical neurons with the DNA methyltransferase inhibitor 5-aza-2-deoxycytidine did not block the downregulation of MAPs by PIWIL1 siRNA (Fig. 6b), suggesting that PIWI may not regulate MAPs through epigenetic modification of target genes, as is the case in Aplysia sensory neurons.

Next, we used quantitative real-time PCR (qPCR) to analyze the decay of mRNAs after transcriptional inhibition (actinomycinD, ActD; 3–6 h) in cultured cortical neurons electroporated with Scramble or PIWIL1 siRNA. After transcription inhibition, PIWIL1 siRNA caused a much faster reduction in the mRNA level of MAP1B (Fig. 6c). This result indicates that PIWIL1 may stabilize the mRNA of MAP1B under normal conditions, which may be one mechanim for PIWIL1’s regulation of cortical radial migration. RNA immunoprecipitation (RIP) was carried out to examine whether PIWIL1 directly interacts with mRNAs of MAP1B. In cortical neurons transfected with flag-tagged HIWI or flag alone, the RNA-protein complex was pulled down using the anti-flag antibody followed by RT-PCR analysis of precipitated mRNAs using MAP1B specific primers. We found that HIWI can specifically associate with the mRNA of MAP1B (Fig. 6d), indicating the stablization of MAP1B mRNA by direct interaction with PIWIL1.

## Discussion

Migrating neurons in the developing brain and sperm cells in testis exhibit a similar polarized morphology. They both experience a polarization process during their morphogenesis. It is likely that critical molecular mechanisms for establishing this polarized morphology are shared by these two distinct cells. Therefore, knowledge about the morphogenesis of either cell may assist us in studying the other. Consistent with this notion, the Lis1 gene, a well-known player in the polarization and migration of cortical neurons, has recently been shown to play similar roles in spermatogenesis. Lis1 mutant spermatids tend to develop several tail-like structures around the nucleus instead of a single tail as seen in normal spermatids [6]. Here we confirmed the expression of an important signaling molecule PIWIL1, which was previously considered to be restrictively expressed in germline tissue, in the developing cerebral cortex. Knocking down PIWIL1 in newborn neurons *in vivo* resulted in defects in their polarization and radial migariton. This is reminiscent of the malformation of sperm in the testis of PIWIL1 knockout mice, where spermatogenesis is arrested at the round spermatid stage without the development of a sperm tail. We found that PIWIL1 may regulate neuronal development through MAPs. Whether regulation of the expression of MAPs by PIWIL1 is also implicated in spermatogenesis remains to be explored.

Lee et al. reported the identification of piRNAs in mouse hippocampus and the expression of MIWI in cultured hippocampal neurons [13]. Recently, Rajasethupathy et al. discovered the expression of piRNAs in the *Aplysia* nervous system and found that the PIWI/piRNA complex could enhance long-term synaptic facilitation by promoting serotonin-dependent methylation of a conserved CpG island in the promoter of CREB2 gene [23]. Contrary to the nucleic localization of PIWI in *Aplysia* sensory neurons, we observed a predominant cytoplasmic localization in neurons, similar to what was observed by Lee et al. in hippocampal neurons [13]. Moreover, since the DNA methyltransferase inhibitor 5-aza-2′-deoxycytidine did not block the downregulation of MAPs by PIWIL1 siRNA (Fig. 6b), PIWI may not regulate MAP expression through methylation of the promoter of target genes in cortical neurons.

Although other possibilities cannot be ruled out, our results clearly support the notion that PIWIL1 may directly bind and stabilize the mRNA of some target genes including MAP1B. This is supported by previous findings that, in spermatocytes, PIWIL1 encodes a cytoplasmic protein which binds mRNAs of ACT (activator of CREM in testis) and CREM target genes and controls their stability [11]. Interestingly, similar mechanisms have been used by another mRNA binding protein, FMRP, which has been reported to control the multipolar/bipolar transition of cortical neurons via binding and regulation of the expression of the mRNA of Cdh2 [24]. Consistent with this mechanism, we observed a cytoplasmic distribution of PIWIL1 in neurons and found that RNA binding domain of PIWIL1 is essential for its function in cortical radial migration.

Recent whole exome sequencing studies show that *de novel* mutations of PIWI family members are strongly associated with autism [12]. Significantly, more retrotransposition events caused by misregulated mobile elements were also reported to occur in autism and other neurological disorders [25, 26]. Considering the known function of PIWI in suppressing transposons [27], another possibility is that PIWIL1 promotes the expression of MAPs indirectly by processing the mRNAs of retrotransposons and suppressing their retrotransposition. Previous studies showed that LINE-1 retrotransposition affects the expression of neuronal genes during neurogenesis [28]. An interesting future question is whether MAPs are indeed the regulatory target of LINE-1 retrotransposons, which are controlled by PIWI and piRNAs.

Our preliminary data showed that there was no clear gross abnormality in the cortical lamination of the PIWIL1 KO mouse, as shown by a normal distribution of Cux1+ neurons in the upper layers and Tbr1+ neurons in the deeper layers (Additional file 8: Figure S6). However, we cannot rule out the possibility that other PIWI family members may compensate PIWIL1 function in the mutant mouse. Since *de novo* mutations in all PIWI family members were discovered in some ASD individuals, it is very likely that these different family members play redundant roles in cortical development. Consistent with this notion, our preliminary data also showed that knockdown of PIWIL2 in rat cortex caused similar migration retardation as PIWIL1 (Additional file 9: Figure S7). Considering that autism is generally regarded as a multi-gene disorder, another possibility is that mutation of a single PIWI family member may not be sufficient to cause a significant developmental abnormality. However, it may generate a sensitive genetic background, which causes more severe developmental defects when associated with other genetic or environmental risk factors. More sophisticated future studies are needed to further address the potential redundant and synergistic functions of different PIWI family members in cortical development.

## Conclusion

In summary, we found that PIWIL1 is expressed in cortical tissues and may promote neuronal polarization and migration through regulating the stability and expression of MAPs. This study discovers a novel function of PIWIL1 in neuronal development and underscores the conserved functions of molecules in the morphogenesis of brain and germline tissue. It also provides a mechanism as to how mutations of PIWI may be linked to develomental brain disorders such as autism.

## Methods

### Animals

Sprague–Dawley rats and C57 mice were provided by SLAC Laboratory Animal Co., Ltd. (Shanghai, China). The PIWIL1 KO mice (B6;129-Piwil1^tm1Hfl^/Mmmh) with a mixed genetic background of 129 and C57BL/6 J were from the Mutant Mouse Regional Resource Center (029995-MU) and were crossed with C57 to maintain the colony. Animals were housed in a standard facility with a 12:12 light–dark cycle. All experimental procedures involving animals were done under the permission of the Bioethics Committee of the Institute of Neuroscience at Chinese Academy of Sciences.

### Constructs

The siRNA sequences were cloned into a pSuper vector. The sequences of PIWIL1 siRNAs are 5′-CAGTCGCGTTTGCGACTGG-3′ (scrambled), 5′-CAAGTAATCGGAAGGACAA-3′ (RNAi1), 5′-GCACAAGGTCACAGAAGTA-3′ (RNAi2), 5′-GCAACAAATTGGACGGAAT-3′ (RNAi3), and 5′-GCAGACTGGTCCAAAGAAA-3′ (RNAi4). Human PIWIL1 and different truncations were generated by PCR amplification and subcloned into the pCAG-IRES-EGFP vector. Mouse MAP2B plasmid was provided by Dr. Jürgen Götz (Queensland Brain Institute, University of Queensland, Australia) and subcloned into the pCAG-IRES-EGFP vector.

### Antibodies

The PIWIL1 antibody for Western blotting (1:1,000) has been described previously [29]. Other antibodies for immunohistochemistry or Western blotting were Tbr2 (rabbit, 1:500; Abcam, Cambridge, UK), green fluorescent protein (GFP) (chicken, 1:1,000; Abcam, Cambridge, UK), BrdU (mouse, 1:200; Sigma, St. Louis, MO), Sox2 (goat, 1:50; Santa Cruz Biotechnology, Santa Cruz, CA), MAP2 (rabbit, 1:1,000; Chemicon, Bioscience Research Reagents, Temecula, CA), Nestin (mouse, 1:100; Millipore, Billerica, MA), doublecortin (rabbit, 1:1,000; Cell Signaling Technology [CST], Danvers, MA), Flag (rabbit, 1: 1,000; Cell Signaling Technology [CST], Danvers, MA), Cux1 (Rabbit, 1:100; Santa Cruz Biotechnology, Santa Cruz, CA), and Tbr1 (Rabbit, 1:500; Abcam, Cambridge, UK).

### *In utero* electroporation (*IUE*)

E16 Sprague–Dawley rats and E14.5 C57 mice were used for *IUE* according to previously reported methods [16, 17]. Timed pregnant rats or mice were anesthetized by intraperitoneal injection of 12 % chloral hydrate (3.5 mL/kg) or 0.7 % sodium pentobarbital (10 mL/kg), respectively. Uteruses were exposed. A mixture of plasmids for siRNA (3 μg/μL for rats and 2 μg/μL for mice) and the enhanced yellow fluorescent protein (EYFP, 3 μg/μL for rats and 1 μg/μL for mice) was injected by trans-uterus pressure microinjection into the lateral ventricle of embryos, using Fast Green (2 mg/mL, Sigma) as an indicator. For rescue experiments, cDNAs in the pCAG-IRES-EGFP vector were mixed and injected with the siRNA plasmid at a molar ratio of 2:1 for HIWI and its truncations or 1:1 for MAP2B. Electric pulses were generated by an electroporator T830 (BTX Molecular Delivery Systems, Holliston, MA) and applied to the cerebral wall at five repeats of 60 V for 50 ms with an interval of 100 ms for rats and 30 V for 50 ms with an interval of 1 s for mice. In each pregnant rat or mouse, embryos in the uterus were randomly selected to be injected with either control or testing plasmids, followed by application of electric pulses of either left or right direction, respectively. After brains were collected, whether the brain was electroporated by control or testing plasmids was judged by which side of the brain had been transfected with the reporter gene. After perfusion, brains were checked under a fluorescent dissecting microscope (Olympus SZX-ZB12) and only those with EYFP expression in the somatosensory cortex were processed for immunohistochemistry analysis.

### Immunohistochemistry

Standard perfusion and fixation procedures were conducted and coronal brain sections of 20 μm were prepared and subjected to standard immunohistochemical analysis. Briefly, sections were permeabilized with 0.2 % Triton X-100 for 30 min and blocked in 5 % bovine serum albumin with 0.2 % Triton X-100 for 1 h at room temperature, followed by incubation with specific primary antibodies at 4 °C overnight. After washing with phosphate-buffered saline for three 10-min periods, sections were incubated with appropriate fluorescence-conjugated secondary antibodies for 1 h at room temperature. Nuclei were labeled by DAPI (4′,6-diamidino-2-phenylindole). For coronal sections from each electroporated brain, 2 slices of the striatum level that exhibited the most intact tissue appearance were chosen for imaging analysis. Images were acquired on a Nikon Neurolucida system or a confocal system (Olympus FV1000 or Nikon A1) and processed using Image-Pro Plus 6.0 (Media Cybernetics, Rockville, MD), ImageJ, and Adobe Photoshop CS 5.0 (San Jose, CA).

### Cell culture and transfection

Cortical tissues of E16 rats were dissected and digested by 0.125 % trypsin. Dissociated neurons were transfected with 6 μg of different plasmids using the Amaxa Nucleofector kit (Lonza, Basel, Switzerland), plated into 35-mm dishes coated with 100 μg/mL poly-d-lysine, and fed with Neurobasal medium supplemented with 10 % fetal bovine serum and 2 % B27.

### PCR

For semi-quantitative RT-PCR, total RNA was extracted from cortical tissues with Trizol reagent (Invitrogen, Carlsbad, CA). About 1 μg of total RNA was converted to cDNA with a RevertAid First Strand cDNA Synthesis kit (Fermentas, Thermo Scientific Molecular Biology, Burlington, ON, Canada), and 1/20 of the cDNA was used in 20-μL PCR reactions. For quantitative real-time PCR (qPCR), total RNA of cells extracted from cultured neurons with Trizol reagent. 1 μg mRNA was used for reverse transcription by PrimeScript RT Master Mix (Takara). SYBR Green Realtime PCR Master Mix (TOYOBO) was used and qPCR was performed using the Rotor-Gene Q machine (QIAGEN). Results were normalized to GAPDH, and data analysis was done by using the comparative C_T_ method in software by QIAGEN.

### RNA binding protein immunoprecipitation assay (RIP)

RNA binding protein immunoprecipitation assay was performed according to a previously published method [30]. Briefly, E16.5 cortical neurons were dissociated and electroporated with CMV-HIWI-3xFlag or CMV-3xFlag. After culturing for 48 h, cells were harvested by polysome lysis buffer (PLB) (100 mM KCl, 5 mM MgCl2, 10 mM HEPES, pH 7.0, 0.5 % NP-40, 1 mM dithiothreitol, 100 U mL-1 RNasin RNase inhibitor [Promega, Madison, WI], 2 mM vanadyl ribonucleoside complex solution, protease inhibitor cocktail). After preclearing, lysates were incubated with anti-flag antibody at 4 °C overnight. After immunoprecipitation, protein was digested in PLB with 0.1 % sodium dodecyl sulfate and 30 μg proteinase K at 50 °C for 30 min. mRNA was isolated and purified using phenol-chloroform-isoamyl alcohol and precipitated by ethanol. Semi-quantitative RT-PCR was then performed to detect bound RNAs with specific primers.

### Statistical analysis and data deposit

All data are presented as mean ± SEM. The normality of data distribution was evaluated by Shapiro-Wilk test in SPSS. Unpaired and two-tailed Student’s *t*-test was used to determine whether significant differences existed between two groups of data. The equality of variance of the two groups of data in comparison was assessed using Levene’s test in SPSS. Equal variance *t*-test was used when *P* > 0.05 was obtained using Levene’s test. Pearson’s correlation algorithms (two-tail) were used to assess the correlation between PIWIL1 levels and the rasiRNA levels. Gene ontology (GO) enrichment analysis was done on http://david.abcc.ncifcrf.gov/. Original data from deep sequencing experiments have been deposited at the GEO database (No. GSE27576 and No. GSE48236).

## Additional files

Additional file 1: Figure S1. Detection of the expression of PIWI and correlation between PIWIL1 and rasiRNA (also known as piRNA) over time. Additional file 2: Figure S2. PIWIL1 knockdown by IUE of siRNAs in mice at E14.5 impaired cortical radial migration, and overexpression of HIWI did not impair the neuronal migration. Additional file 3: Figure S3. Western blots verified the expression of mouse D633A mutant of PIWIL1 and the RNAi 2-resistant D633A mutant (D633A-Res). Additional file 4: Figure S4. The morphology of radial glial fibers were not affected by PIWIL1 knockdown. Additional file 5: Figure S5. PIWIL1 didn’t play a major regulatory role in proliferation and differentiation of neural progenitor cells. Additional file 6: Table S1. Top 10 GO terms for the 667 genes downregulated upon PIWIL1 knockdown. Additional file 7: Table S2. Top 10 GO terms for the 286 genes upregulated upon PIWIL1 knockdown. Additional file 8: Figure S6. Normal distribution of Cux1 and Tbr1 neurons in the cortex of P3 PIWIL1 KO mice. Additional file 9: Figure S7. PIWIL2 knockdown by IUE of siRNA in mice impaired cortical radial migration.
