# Erratum to: Novel function of PIWIL1 in neuronal polarization and migration via regulation of microtubule-associated proteins

**DOI:** 10.1186/s13041-016-0201-y

**Published:** 2016-02-23

**Authors:** Ping-ping Zhao, Mao-jin Yao, Si-yuan Chang, Lan-tao Gou, Mo-fang Liu, Zi-long Qiu, Xiao-bing Yuan

**Affiliations:** Institute of Neuroscience and State Key Laboratory of Neuroscience, Shanghai Institutes for Biological Sciences, Chinese Academy of Sciences, Shanghai, 200031 China; Graduate School of Chinese Academy of Sciences, Shanghai, 200031 China; State Key Laboratory of Molecular Biology, Institute of Biochemistry and Cell Biology, Shanghai Institutes for Biological Sciences, Chinese Academy of Sciences, Shanghai, 200031 China; Current Affiliation: Hussman Institute for Autism, Baltimore, MD 21201 USA

After publication of this article [1], the authors noticed two errors in Figs. 2e and 3e.

**Fig. 2:**
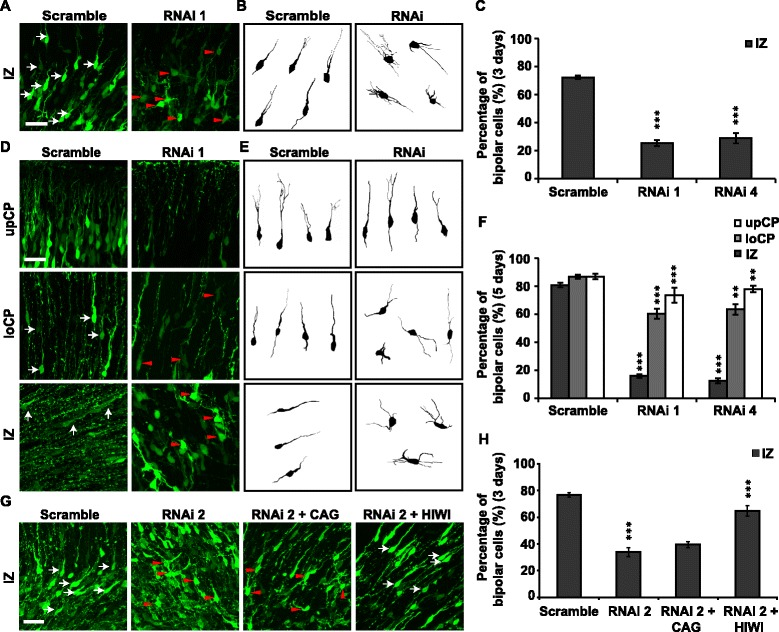
PIWIL1 is required for the multipolar–bipolar transition of postmitotic neurons. **a**, **d** Morphology of labeled neurons in different cortical regions 3 or 5 days post-IUE with siRNA 1. **b**, **e** Traces of labeled neurons 3 or 5 days after IUE respectively. **c**, **f** Percentage of bipolar cells (white arrows) in different cortical regions. Data are from at least 3 independent IUE experiments. **g** Typical morphology of labeled mouse neurons in the IZ 3 days after IUE with RNAi 2 or RNAi 2 plus HIWI compared with individual control plasmid. **h** Percentage of bipolar cells at the IZ of electroporated mouse cortex. Scale bar, 30 μm. Error bar, SEM, **P < 0.01, ***P < 0.001 (Student’s t-test)

**Fig. 3:**
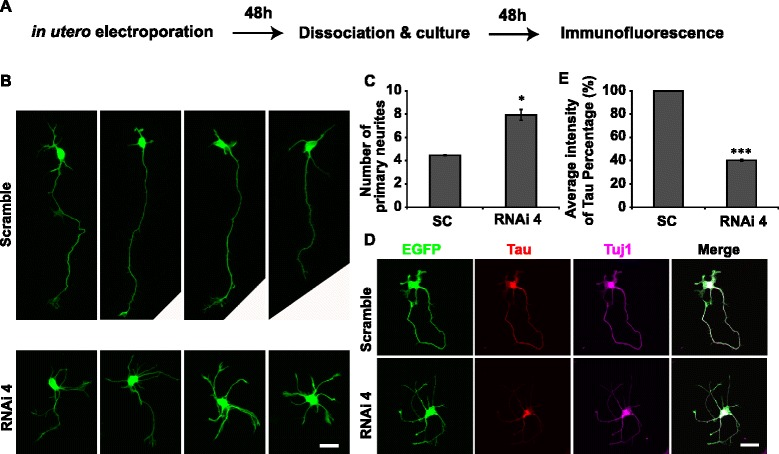
PIWIL1 knockdown impairs polarization of cortical neurons ex vivo. **a** Diagram of the ex vivo assay. **b**, **c** Average numbers of primary neurites of electroporated cells. **d** Immunostaining: cultured neurons with PIWIL1 knockdown exhibited multipolar morphology and lower levels of Tau but not Tuj1. **e** Average neurites’ fluorescence intensity of Tau in GFP+ neurons. Scale bar, 20 μm. Error bar, SEM, *P < 0.05, ***P < 0.001 (Student’s t-test)

The images provided for Fig. 2e in squares ‘Scramble upCP’ and ‘Scramble IoCP’ were incorrect. The correct version of Fig. 2 is included in this erratum.

In Fig. 3e, the label ‘RNAi 4’ was missing from the x axis. The correct version of Fig. 3 is also included in this erratum.
