# Supplementary figures and images for: Novel function of PIWIL1 in neuronal polarization and migration via regulation of microtubule-associated proteins
Source: Mol Brain. 2015 Jun 24;8:39. doi: 10.1186/s13041-015-0131-0 (PMC4477296; doi:10.1186/s13041-015-0131-0)

# E14.5 mouse

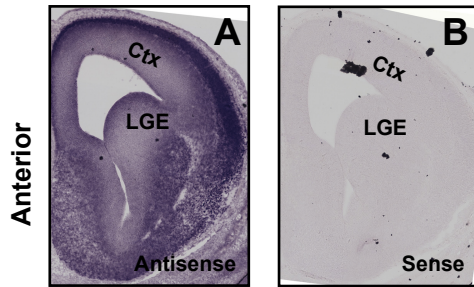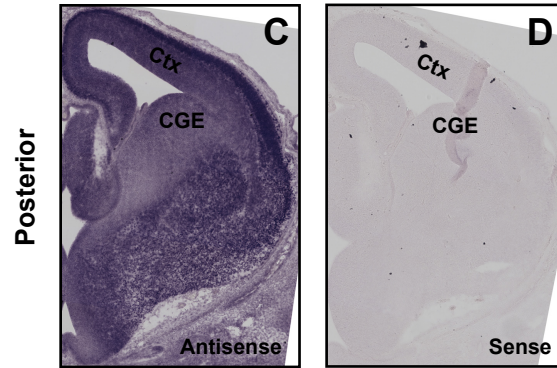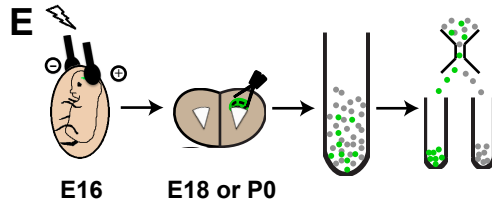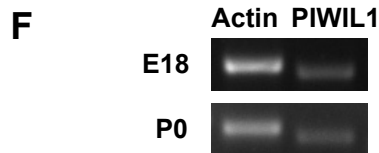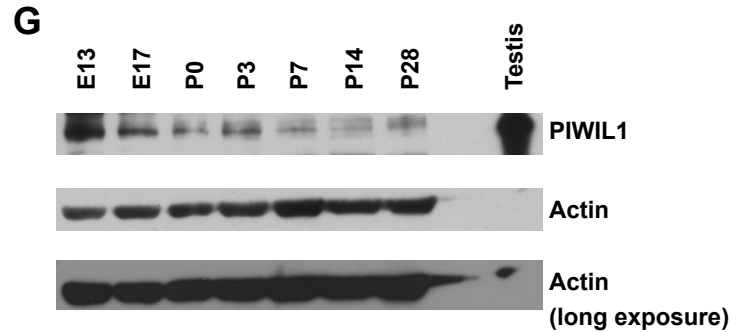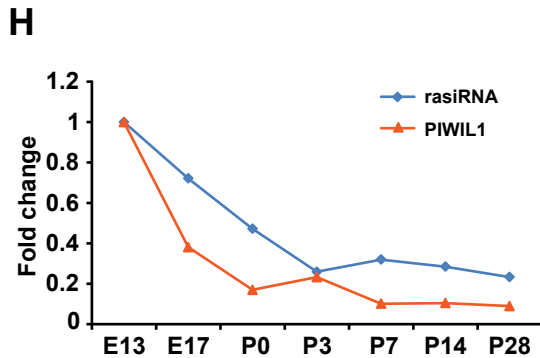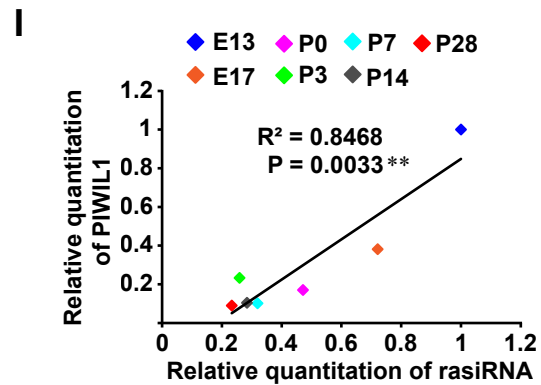

Supplement: Additional file 1: Figure S1. — Detection of the expression of PIWI and correlation between PIWIL1 and rasiRNA (also known as piRNA) over time. [file 13041_2015_131_MOESM1_ESM.pdf]

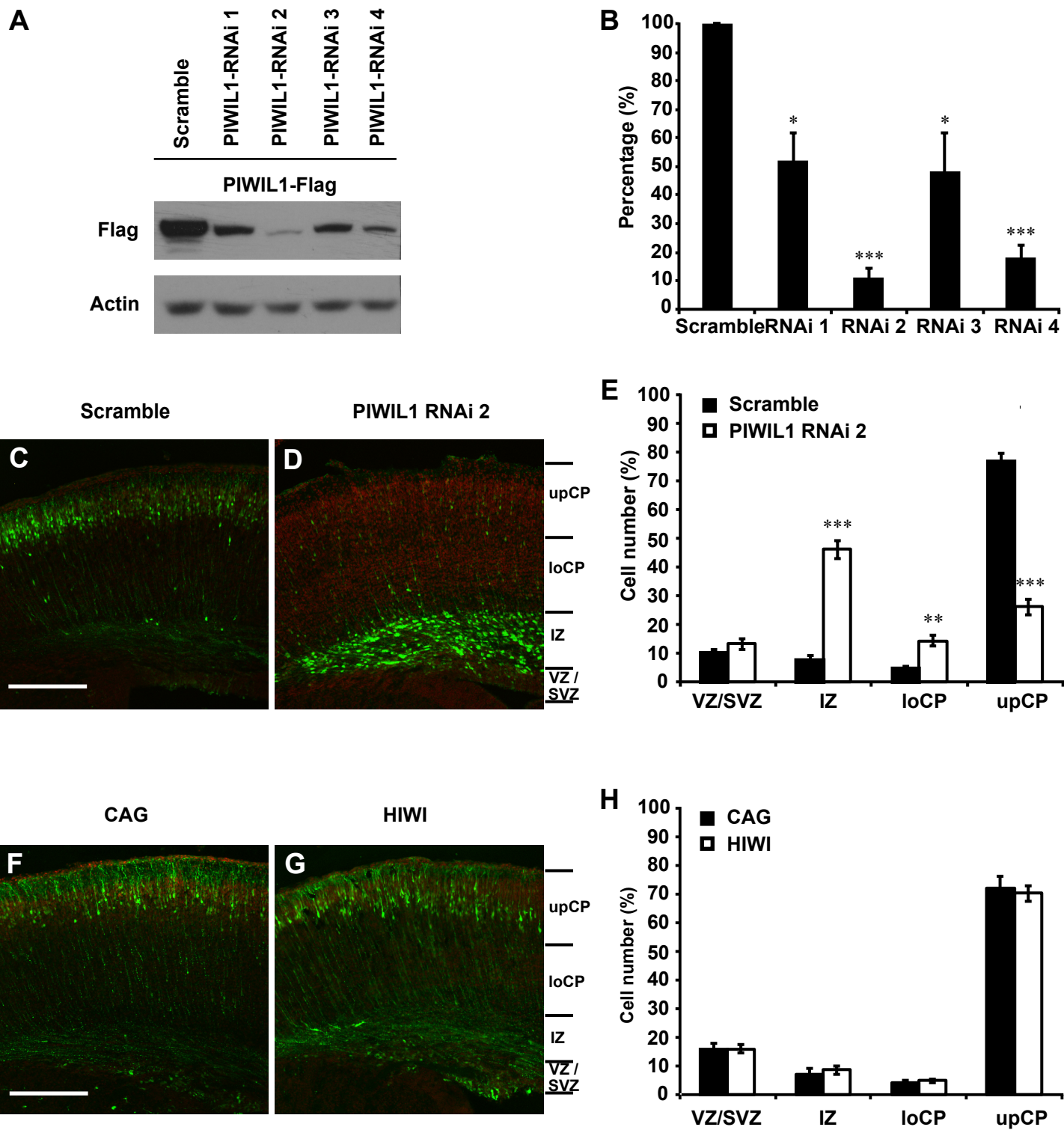

Supplement: Additional file 2: Figure S2. — PIWIL1 knockdown by IUE of siRNAs in mice at E14.5 impaired cortical radial migration, and overexpression of HIWI did not impair the neuronal migration. [file 13041_2015_131_MOESM2_ESM.pdf]

**A**

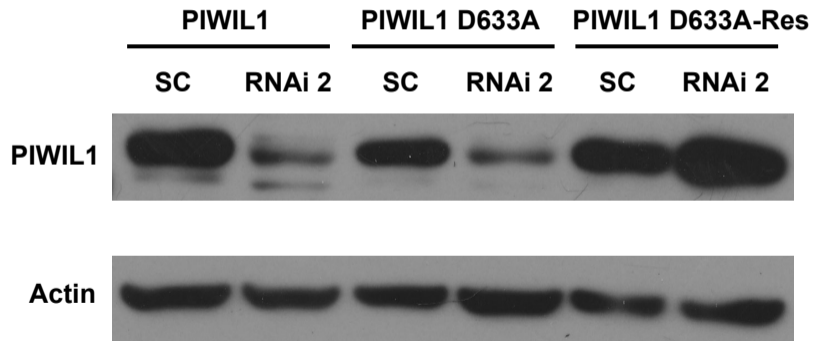

Supplement: Additional file 3: Figure S3. — Western blots verified the expression of mouse D633A mutant of PIWIL1 and the RNAi 2-resistant D633A mutant (D633A-Res). [file 13041_2015_131_MOESM3_ESM.pdf]

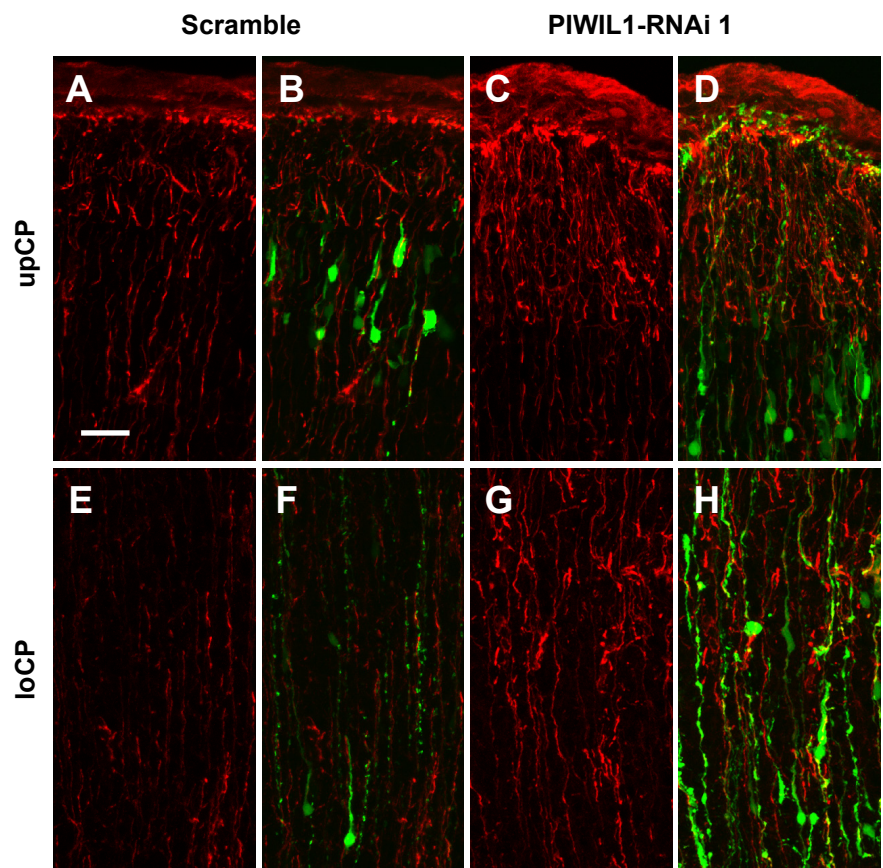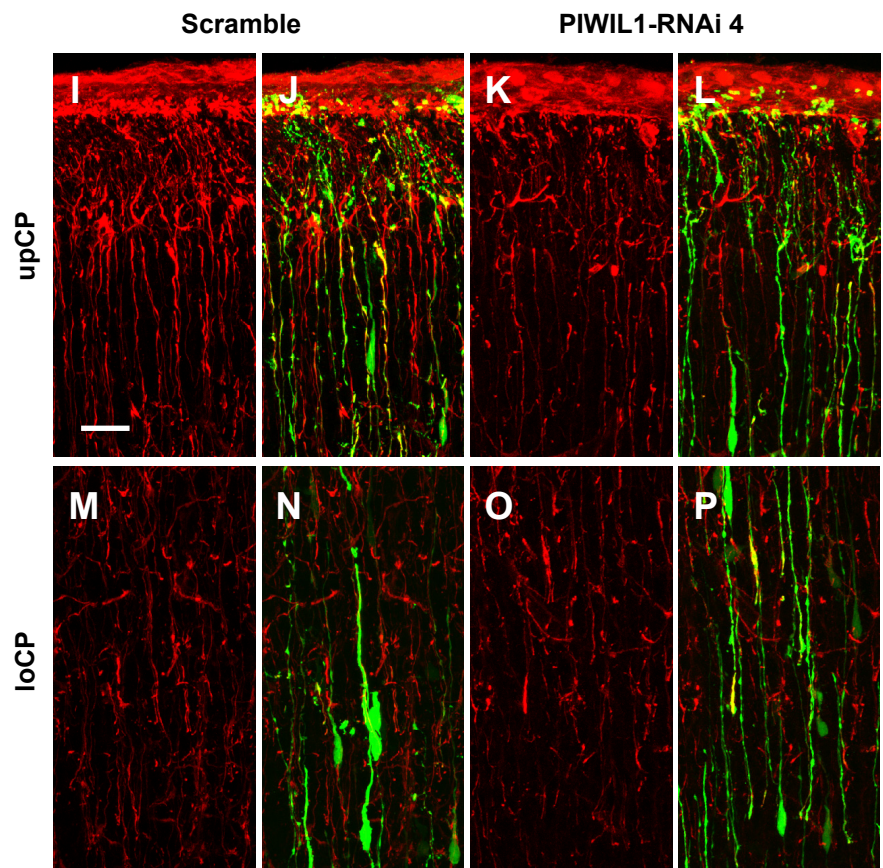

GFP Nestin

Supplement: Additional file 4: Figure S4. — The morphology of radial glial fibers were not affected by PIWIL1 knockdown. [file 13041_2015_131_MOESM4_ESM.pdf]

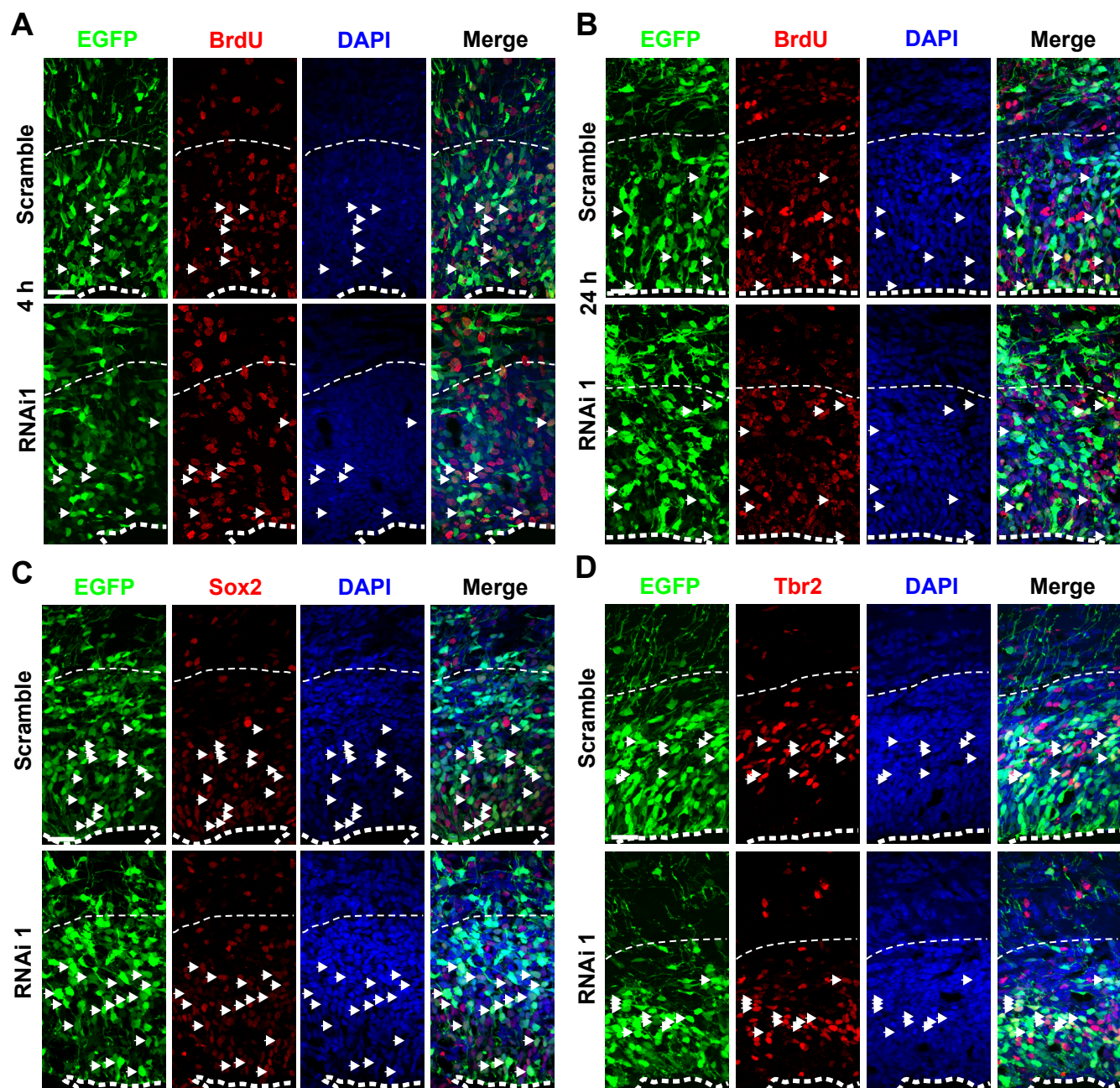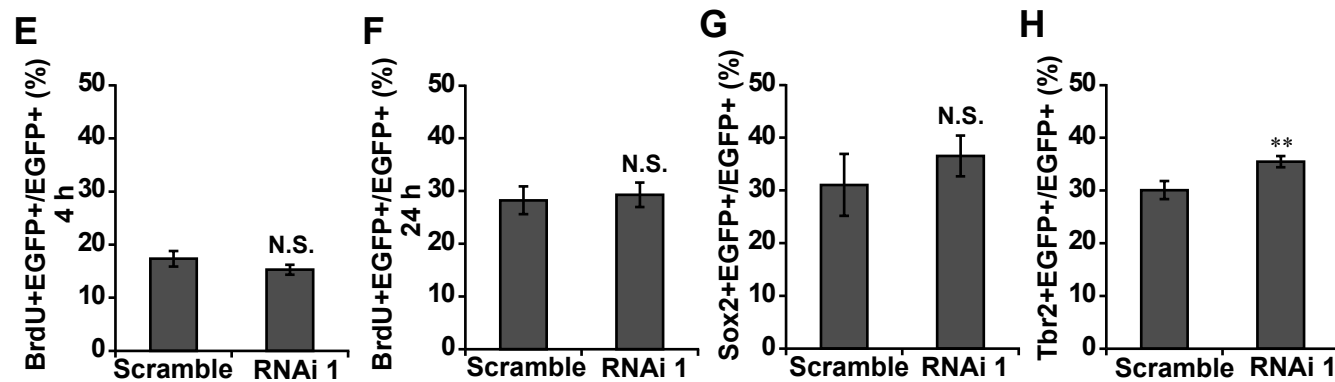

Supplement: Additional file 5: Figure S5. — PIWIL1 didn’t play a major regulatory role in proliferation and differentiation of neural progenitor cells. [file 13041_2015_131_MOESM5_ESM.pdf]

**A****P3 somatosensory cortex**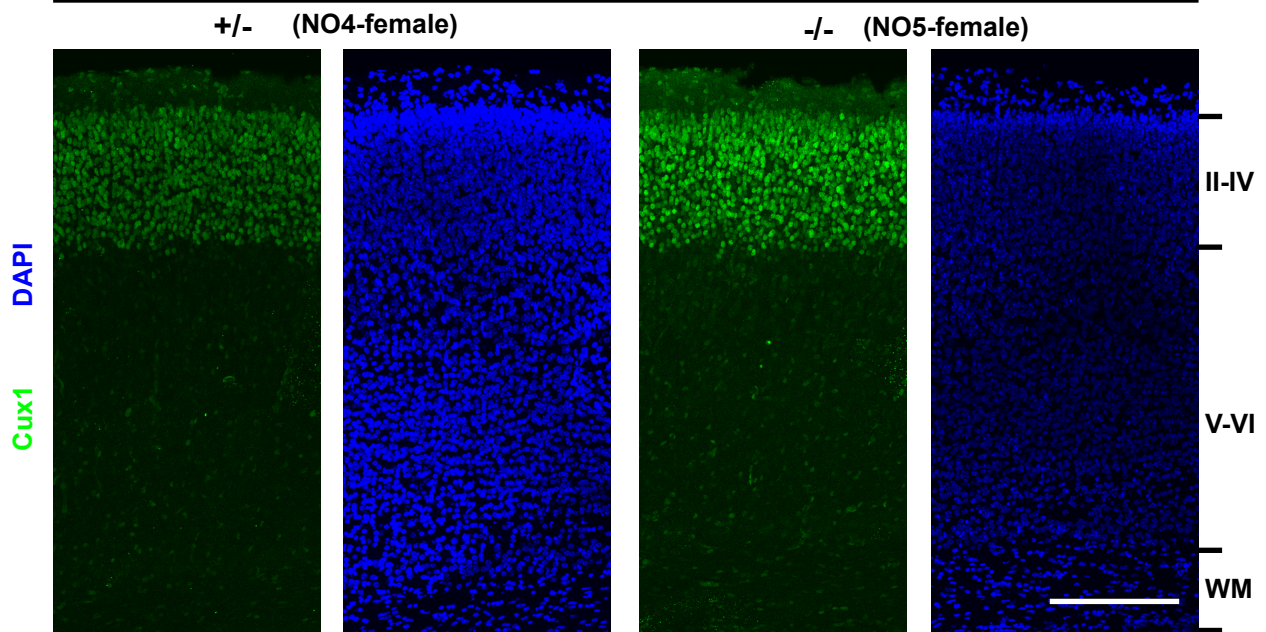**B****P3 somatosensory cortex**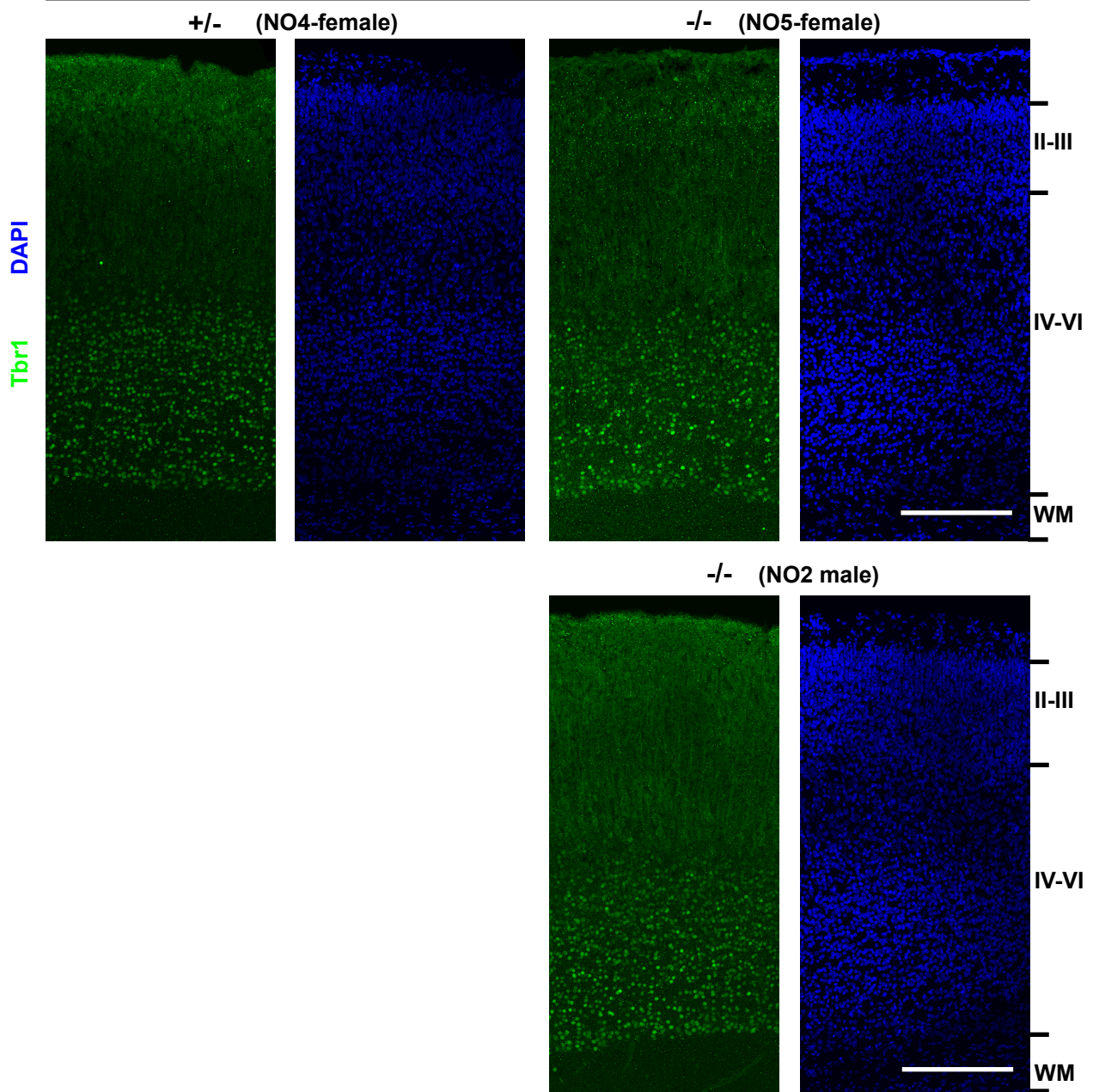

Supplement: Additional file 8: Figure S6. — Normal distribution of Cux1 and Tbr1 neurons in the cortex of P3 PIWIL1 KO mice. [file 13041_2015_131_MOESM8_ESM.pdf]

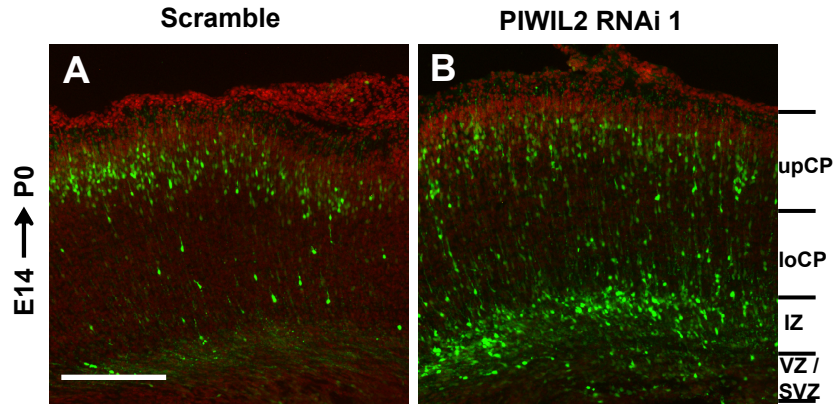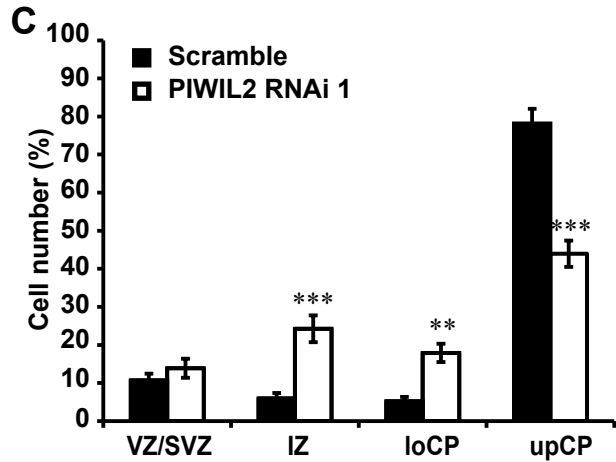

Supplement: Additional file 9: Figure S7. — PIWIL2 knockdown by IUE of siRNA in mice impaired cortical radial migration. [file 13041_2015_131_MOESM9_ESM.pdf]
